# Visual appearance of blood vessels: a phantom study

**DOI:** 10.1364/BOE.579246

**Published:** 2026-01-29

**Authors:** Markus Wagner, David Hevisov, Corinna Konrad, Hannes Schmidt, Florian Foschum, Alwin Kienle

**Affiliations:** 1 Faculty of Natural Sciences, Ulm University, D-89081 Ulm, Germany; 2 Institute for Laser Technologies in Medicine and Metrology at the University of Ulm, Helmholtzstr. 12, D-89081 Ulm, Germany

## Abstract

The blue appearance of veins has been addressed in previous studies; however, an experimental demonstration that reproduces this phenomenon with comprehensible optical properties has not yet been achieved. To address this gap, silicone-based skin phantoms were fabricated to replicate the optical properties of human skin. Cylindrical vessel inclusions of varying depths and diameters were incorporated into the phantoms and filled with silicone mimicking blood at different oxygenation states. The vessels’ appearance was quantitatively evaluated using calibrated photography and hyperspectral imaging. Monte Carlo light transport simulations were employed to support the experimental findings. The phantoms with venous inclusions exhibited the characteristic blue hue of veins, thus demonstrating that vein blueness does not arise solely from blood’s intrinsic absorption but from the complex interplay of tissue optics and visual perception. These experimental findings support our previously proposed theory.

## 1. Introduction

Although blue blood is found in some animal species, such as horseshoe crabs and certain mollusks, human blood is always red - regardless of its oxygenation state [1]. Yet, when we observe our veins through the skin, they often appear bluish. This intriguing phenomenon has fascinated scientists and physicians for centuries and remains a subject of active research and debate in biomedical optics [2–9]. Our theory suggests that veins appear blue due to wavelength-dependent light–tissue interactions and human color perception [2]. Red light, with its longer wavelength, penetrates deeper into tissue and is absorbed to a relatively high degree by blood in veins, reducing red reflectance above the vein. In contrast, blue light is mostly scattered or absorbed in the superficial skin layers and interacts less with the vein (although the absorption coefficient for blue light is much larger than for red light). Therefore, the reflection above the vein is less diminished. Compared to the surrounding tissue, this results in a greater relative reduction of red light than blue light above the vein. Brain mechanisms of color perception, as described by Retinex theory, interpret the spectral reflectance contrast relative to the surrounding tissue, leading to the perception of veins as blue despite the reflectance above the vein may contain more red light [2]. To investigate this effect, we performed Monte Carlo simulations modeling the influence of blood vessel depth, diameter and blood oxygenation on visual appearance [6]. However, to date, there has been no experimental validation of how these parameters affect the observed color of blood vessels.

To better understand the visual appearance of blood vessels, both computational simulations and experimental models are invaluable. Monte Carlo simulations enable detailed modeling of the complex interactions of light with biological tissues [10], allowing systematic exploration of parameters such as blood vessel depth, diameter and blood oxygenation [6]. In parallel, optical phantoms - real objects that mimic the optical properties of skin and blood - facilitate controlled experimental investigations and validation of simulation results [2]. Accurately mimicking human tissue requires a thorough understanding of the optical characteristics of skin including the subcutaneous fat. Human skin scatters and absorbs light due to various components, including collagen and elastin fibers. The degree of scattering depends on the composition and size of these microstructures, while absorption in the visible spectrum is predominantly influenced by blood and melanin [11–13]. By adding scattering particles or absorbing media to phantom materials such as silicone or epoxy resin, their optical properties can be tuned to closely match those of real tissues [14–16].

The aim of this work is to systematically investigate the color perception of blood vessels using silicone-based optical phantoms. By replicating the optical properties of human tissue and blood and varying vessel characteristics such as depth, diameter and oxygenation, we seek to support our hypothesis of the underlying mechanisms responsible for the bluish appearance of veins and to identify the conditions under which arteries may also become visible. Physics-based Monte Carlo simulations were used to support the experimental findings. This approach not only advances our understanding of a classic question in biomedical optics but also has implications for medical diagnostics and imaging technologies.

## 2. Materials and methods

### 2.1. Fabrication of silicone phantoms

**Fig. 1.**
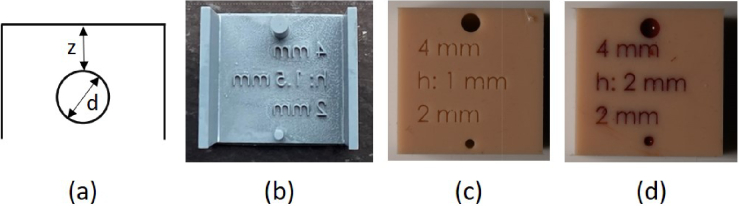
(a) Schematic of the phantom geometry, illustrating the vessel diameter *d* and depth *z*. (b) 3D-printed mold containing vessels (cylindrical inclusions) for the base material. (c) Example of the silicone phantom after curing in the 3D-printed mold. (d) Phantom after being filled with silicone mimicking blood.

For the preparation of tissue phantoms, Elastosil M 4641 A/B (Wacker Chemie AG, Germany) was used as the silicone base. The fabrication process involved several mixing steps, utilizing a dual asymmetric vacuum centrifugal mixer (SpeedMixer DAC 800, Hauschild Engineering, Germany) to ensure thorough dispersion of pigments and uniform optical properties. All mixing was performed under vacuum to prevent air bubble formation and to maintain consistent material quality. A comprehensive and detailed description of this production process is provided by us [17]. To characterize the optical properties, layer samples (slabs) of various thicknesses (1, 2, 4 and 6 mm) and a diameter of 35 mm were produced. These phantoms were fabricated using two acrylic plates and a custom 3D-printed spacer. After curing, the slabs were removed from the mold and used to determine the optical properties of the phantom. For vessel-mimicking phantoms, we designed cylindrical channels with diameters *d* of 2 and 4 mm, positioned at various depths *z* of 0.5, 1, 1.5 and 2 mm beneath the surface, as schematically illustrated in Fig. 1(a). These phantoms were created by fabricating negative molds using stereolithography (SLA) 3D printing (Sonic Mighty Revo, Phrozen, Taiwan), as shown in Fig. 1(b). The molds were placed on an acrylic plate to ensure a flat surface during casting. The skin-like pigmented silicone was poured into the molds and allowed to cure. After curing, the mold plastic within the vessel channels was carefully removed and replaced with silicone mixtures containing different pigment concentrations, simulating blood with varying oxygenation levels. This two-stage casting approach enabled integration of vessel structures within the phantom matrix. An example phantom is shown in Fig. 1(c,d). To adapt the scattering properties, zirconium oxide (ZrO_2_) nanoparticles (Zirconium Powder 200 nm, US Research Nanomaterials, USA) were incorporated. For absorption tuning, a library of absorption spectra was established, comprising 15 organic and inorganic pigments. Only organic pigments with a blue wool scale rating of at least 7 and inorganic pigments with a rating of 8 were selected to ensure high resistance to fading. Each pigment was initially dispersed in silicone to create concentrated stock samples, whose absorption spectra were then measured. Seven pigments were finally selected for use in the phantoms, see Tab. S1 and Fig. S1. The optimal concentrations of these pigments were determined by fitting the measured absorption spectra to target absorption using the lsqcurvefit function in MATLAB 2023a [18]. The fitting process involved iterative adjustment of pigment concentrations to minimize the deviation between measured and modeled spectra. To ensure robustness and avoid convergence to local minima, the optimization was repeated 30 times with randomized initial parameters. The set of concentrations yielding the lowest chi-squared error was selected for phantom fabrication.

### 2.2. Optical characterization and inverse modeling

The absorption coefficient $\left(\mu_{\text{a}}\right)$ and the reduced scattering coefficient $\left(\mu_{\text{s}}^{\prime }\right)$ of the silicone phantoms were determined from measurements of diffuse reflectance (*R*) and transmittance (*T*) using a custom integrating sphere setup, as previously described by Foschum et al. and Bergmann et al. [19,20]. The system is a laboratory setup of the SphereSpectro 150H (Gigahertz Optik GmbH, Germany), featuring a 3D-printed, barium sulfate-coated sphere with a 150 mm internal diameter. Measurements were conducted over a spectral range from 400 nm to 1550 nm. To extract $\mu_{\text{a}}$ and $\mu_{\text{s}}^{\prime }$ from the measured *R* and *T*, a combination of Monte Carlo simulations and analytical model of light propagation in the sphere including ports was employed. The evaluation assumed a Henyey-Greenstein phase function with an asymmetry parameter g=0.7 , appropriate for the optically thick samples used in this study. The refractive index of the silicone was determined by spectroscopic ellipsometry (SENresearch 4.0, SENTECH, Germany), revealing a wavelength-dependent index between 1.43 and 1.40 in the visible range. All measurements were performed in triplicate to ensure reproducibility. For comparison, the optical properties of human forearm skin were also measured using the same integrating sphere setup. In this case, a new look-up table (LUT) was generated by modeling the forearm as a volume that can be approximated as a quasi-semi-infinite medium in the Monte Carlo simulation. Since only reflectance could be measured (not transmittance), the evaluation was adapted to estimate $\mu_{\text{a}}$ using a predefined, wavelength-dependent $\mu_{\text{s}}^{\prime }$ , either from known scatterers or literature values. The LUT was then used to derive $\mu_{\text{a}}$ from the measured *R* and the predefined $\mu_{\text{s}}^{\prime }$ .

### 2.3. Photo box

To obtain calibrated images of the vessel phantoms, a custom-built photo box was employed, as published by Hevisov et al. [21]. The interior was lined with low-reflectance black fabric to minimize stray light. The imaging system included a rotatable multi-axis mount constructed from aluminum profiles, allowing flexible positioning of samples and camera. A Nikon D7500 DSLR camera with a 60 mm AF-S Micro Nikkor lens was mounted perpendicular to the sample surface. Illumination was provided by a custom LED white light source positioned at an angle of 45^∘^ to the left above the surface normal. An aperture in front of the light source restricted the illuminated area to the region of interest, further reducing unwanted stray light.

### 2.4. Monte Carlo simulations and color analysis

A tetrahedron-based Monte Carlo simulation, a stochastical solution of radiative transport equation, was used to model light propagation in tissue and vessels and therefore the visual appearance of blood vessels, as detailed by Hevisov et al. [21]. For each wavelength from 400 to 700 nm (in 5 nm steps), approximately 1 × 10^9^ photons were traced in reverse from the camera through the scene, without relying on simplified reflectance models, enabling a realistic representation of scattering and absorption in tissue. The simulation incorporates the actual geometry, including the light source and camera, spectral characteristics of the setup as well as the optical properties of the phantom and is implemented on a Graphics Processing Unit for computational efficiency. For colorimetric analysis, both the simulated spectra and the real camera images were initially represented in the camera’s RGB color space (i.e., as raw images). To achieve this for the simulated spectra, we weighted the spectra by the wavelength-dependent camera sensitivity of the corresponding RGB channel and the spectral power distribution of the light source, thereby generating RGB values that correspond to the camera’s raw response under the given illumination conditions. These RGB values were then converted to the XYZ color space using appropriate, camera-specific color conversion matrices. Subsequently, the XYZ values were either transformed into the L*a*b* color space to caluclate Δ*E* or into the sRGB color space for visualization. As demonstrated previously, our method achieves a color difference (Δ*E*) of less than 1 between simulated and real photographs, indicating that no visually perceptible color deviations occur [21].

### 2.5. Hyperspectral imaging

Hyperspectral images were acquired using an Ultris S5 camera (Cubert GmbH, Germany). The camera was integrated into a setup comprising a halogen light source (Flexilux 600 Longlife, Schölly Fiberoptic GmbH, Germany). After collimation, the light was directed to a digital micromirror device (V4100 Controller Board, ViALUX Messtechnik + Bildverarbeitung GmbH, Germany), which redirected the illumination through a telecentric lens (TC2MHRP036-C, Opto Engineering, Italy) onto the sample, ensuring uniform illumination. The reflected light was then captured by the camera positioned at 20^∘^ relative to the sample normal. Images were recorded across the wavelength range of 450–850 nm, with a spectral resolution of 8 nm.

## 3. Results

### 3.1. Optical properties of the skin mimicking phantom

The goal of this study was to develop a homogeneous optical phantom whose reflectance and underlying absorption coefficient and reduced scattering coefficient realistically match those of human forearm tissue within the visible wavelength range (400–700 nm). This enables a tissue-mimicking color appearance and comparable light propagation characteristics to those found in human tissue.

**Fig. 2.**
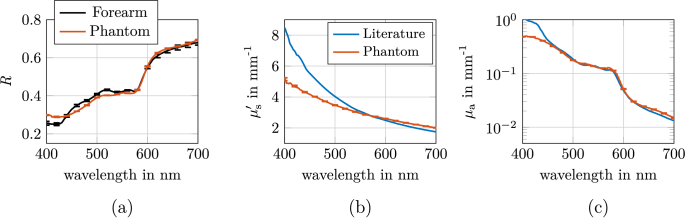
(a) Reflectance measured with an integrating sphere for a human forearm and for the produced phantom designed to mimic the forearm’s reflectance. (b) The reduced scattering coefficient from a representative literature value for porcine skin by Bergmann et al. [22] (blue line) and that of the produced phantom (orange line). (c) The absorption coefficient of the phantom (orange line) and the theoretical absorption coefficient corresponding to the blue $\mu_{\text{s}}^{\prime }$ curve in (b) (blue line), which was used to match the forearm reflectance.

To determine the target values, the reflectance of the inner forearm of a healthy 30-year-old volunteer (Fitzpatrick skin type I) was measured using an integrating sphere setup, see Fig. 2(a). Measurements were taken three times at a hairless site to ensure reproducibility. This anatomical region was specifically chosen to provide representative optical parameters for human skin. The phantom was designed as a homogeneous material and does not replicate the multilayered structure of real skin. A model assuming a homogeneous quasi-semi-infinite medium was used to determine the optical properties from the measured reflectance spectra, as described in Sec. 2.2. Since no transmission data were measurable, the reduced scattering coefficient was predefined - either according to literature values for porcine skin or set by adjusting the concentration of zirconium dioxide particles so that $\mu_{\text{s}}^{\prime }$ at 550 nm matched the porcine skin, as shown in Fig. S2(b) [22]. Although various $\mu_{\text{s}}^{\prime }$ values, both higher and lower, are described in the literature for various tissue types, the values chosen here provide a robust foundation for the present phantom study [11, 23, 24]. Using the predefined $\mu_{\text{s}}^{\prime }$ and the given *R*, the absorption coefficient was calculated across the entire spectral range, see Fig. S2(c).

These values for $\mu_{\text{a}}$ and $\mu_{\text{s}}^{\prime }$ served as the basis for phantom fabrication. To accurately replicate the desired absorption spectrum, seven specific dyes were selected, see Tab. S1, and their concentrations were optimized, following the procedure outlined in Sec. 2.1, to match the target absorption. The fit results are shown in Fig. S3(a). The phantom was subsequently fabricated by precisely weighing and mixing the calculated amounts of each pigment. Following production, the optical properties $\mu_{\text{a}}$ and $\mu_{\text{s}}^{\prime }$ of the phantom were determined and are shown in Fig. 2. The phantom closely matched the target reflectance, with deviations well below 10% between 440 and 700 nm. In the wavelength range between 400 and 440 nm, a larger deviation between the two reflectances was observed, as the absorption could not be matched in this region as no suitable pigment was available. The reduced scattering coefficient also closely matched the literature values between 500 and 700 nm. Although the ZrO_2_ particles used show the steepest increase in scattering with wavelength among the ten considered and characterized scattering particles, their increase in $\mu_{\text{s}}^{\prime }$ at shorter wavelengths was less pronounced than expected for tissue. Minor deviations between the calculated and realized optical properties are due to unavoidable measurement uncertainties and slight inaccuracies in weighing the components. However, these deviations are within the range of experimental tolerance and do not compromise the phantom’s suitability as a tissue mimicking material.

### 3.2. Optical properties of the blood mimicking phantom

**Fig. 3.**
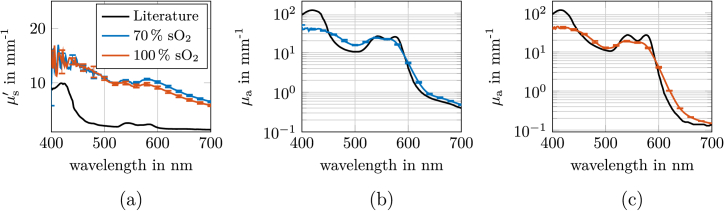
(a) Reduced scattering coefficient of the venous and arterial phantoms (blue and orange lines, representing 70 % and 100 % oxygenation, respectively) compared to reference blood data (black line, 45 % hematocrit). The phantoms exhibit higher $\mu_{\text{s}}^{\prime }$ values than physiological blood, which is attributed to the intrinsic scattering properties of the organic and inorganic pigments used. (b) Absorption coefficient of the 70 % oxygenation phantom (venous) compared to reference blood data (black line, 45 % hematocrit). (c) Absorption coefficient of the 100 % oxygenation phantom (arterial) compared to reference blood data (black line, 45 % hematocrit).

To realistically simulate blood vessels, we fabricated silicone phantoms mimicking blood representing both venous and arterial blood. For veins, an oxygenation level of 70 % was assumed, while for arteries, 100 % oxygenation was used. These values reflect typical physiological conditions [25–27]. The target optical properties were defined based on an extensive literature review. Absorption and reduced scattering coefficients were primarily taken from the review by Bosschaart et al., which provides wavelength-resolved data for whole blood adjusted to a hematocrit of 45 % [28]. The reduced scattering coefficient was further informed by measurements from Friebel et al. [29]. To replicate the absorption spectra of blood, we employed the pigments described in Fig. S1 and Tab. S1. The concentrations of each pigment were determined by fitting the combined absorption spectra to the target values for both oxygenation states, as described in Sec. 2.1. The results for the fit are shown in Fig. S3(b) and (c). This approach enabled us to closely approximate the absorption characteristics of blood across the visible spectrum. Phantoms were fabricated by thoroughly mixing the calculated pigment concentrations into a silicone matrix. To ensure reliable determination of $\mu_{\text{a}}$ and $\mu_{\text{s}}^{\prime }$ , slabs of three different thicknesses (approximately 60 µm, 0.5 mm and 1 mm) were produced for each oxygenation state. The optical properties $\mu_{\text{a}}$ and $\mu_{\text{s}}^{\prime }$ were determined using an integrating sphere setup (see Sec. 2.2). For the thicker slabs (0.5 mm and 1 mm), reliable determination of optical properties was limited to wavelengths above approximately 620 nm due to the high absorption of blood. However, the results from all three thicknesses were in excellent agreement in their respective valid wavelength regions, supporting the robustness of the measurement approach. The trustworthiness of the absorption measurements in the thin slabs was further validated by comparing predicted and measured absorption curves for diluted samples, which showed good agreement. Figure 3 shows the results and compares the phantoms and literature values. Panel (a) depicts the reduced scattering coefficient of the phantoms (blue and orange lines for 70 % and 100 % oxygenation, respectively), which is compared to the reference data for blood (black line, 45 % hematocrit and 100 % oxygenation). The phantoms exhibit higher $\mu_{\text{s}}^{\prime }$ values than physiological blood, attributable to the intrinsic scattering of the organic and inorganic pigments used. This elevated scattering is a trade-off required to achieve the high absorption necessary for blood-mimicking phantoms. Panel (b) illustrates the absorption coefficient for the 70 % oxygenation phantom compared to the reference data for blood (black line, 45 % hematocrit), whereas Panel (c) presents the absorption coefficient for the 100% oxygenation phantom compared to the reference data for blood (black line, 45 % hematocrit). The absorption spectra of the phantoms closely match the target values between 450 and 700 nm. The Soret band peak at approximately 430 nm, however, could not be fully reproduced due to limitations in the available pigments.

### 3.3. Simulative assessment of color differences between phantom and literature values

**Fig. 4.**
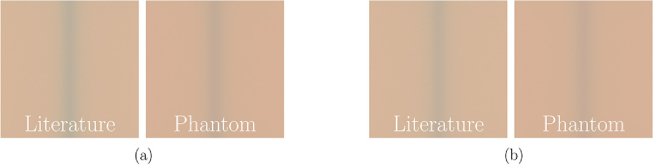
Comparison of simulation results for a blood vessel with *d* = 2 mm and *z* = 1 mm, using literature-derived and produced phantom optical properties from Fig. 2 and Fig. 3, at (a) 70 % and (b) 100 % vessel oxygenation, respectively.

Since the absorption and reduced scattering coefficients of the phantoms could not be perfectly matched to those found in human tissue, as detailed in Fig. 2 and Fig. 3, we employed Monte Carlo simulations, described in Sec. 2.4, to quantitatively assess the resulting color differences under controlled illumination and detection conditions. For this purpose, a cubic tissue model with an edge length of 30 mm was used incorporating cylindrical inclusions of diameter *d* and a depth *z*, as illustrated in Fig. 4(a). The illumination was provided by two rectangular light sources (each 160 mm × 160 mm) positioned symmetrically to the left and right of the sample. The distance from the center of each light source to the center of the object surface was 270 mm. Both light sources were oriented at 45^∘^ relative to the sample surface, ensuring uniform and symmetric illumination. Detection was performed orthogonally above the sample surface using a pinhole camera setup, with the detector aligned parallel to the sample surface. For the phase function, we assumed a Henyey-Greenstein function. Regarding the phantoms, a *g*-factor of 0.7 for both blood and tissue was assumed. Regarding the literature values, we set the *g*-factor for tissue to 0.9, while for blood, the *g*-factor was adopted from the literature values reported by Bosschaart et al. [28,30]. The refractive index for the literature values was set to 1.4 for all wavelengths in the MC simulations. For the phantom, we used the wavelength-dependent refractive index of silicone as measured by [17]. The phantom color was simulated by converting the calculated reflectance spectra into RGB values, as described in Sec. 2.4. To enable direct comparison of the simulated images, all images were normalized to the maximum intensity across all simulations. This normalization ensures that the simulated images are comparable in terms of brightness, allowing differences in color to be attributed to the optical properties. Figure 4(a) and (b) presents a comparison of the resulting color appearance of the literature and the phantom values for an inclusion with *d* = 2 mm and *z* = 1 mm, parameters that fall within the typical range of visible veins [31,32]. Panel (a) compares the color of a vessel containing 70% oxygenated blood, while panel (b) shows the color of an artery with fully oxygenated blood (100%). Panel (a) reveal that the characteristic bluish hue of veins is faithfully reproduced by the phantom, although minor deviations in hue and saturation remain. Panel (b) shows that also arteries, fully oxygenated blood, could be visible in the phantom evaluation. Between the phantom and the literature tissue color, a Δ*E* of 1.7 was observed, indicating that the phantom accurately represents the color of the assumed literature values, as a Δ*E* of 1.7 is barely perceptible to the human eye.

### 3.4. Simulative assessment of impact of blood optical properties on color perception

**Fig. 5.**
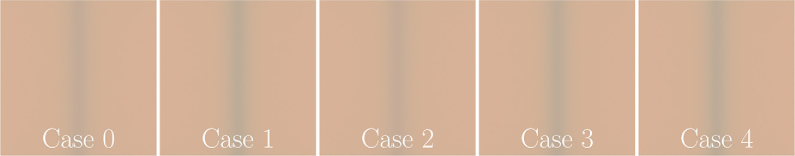
Simulated color impressions for different combinations of $\mu_{\text{a}}$ and $\mu_{\text{s}}^{\prime }$ of venous blood (*d* = 2 mm and *z* = 1 mm): Case 0 (the real phantom): $\mu_{\text{a}}$ : phantom, $\mu_{\text{s}}^{\prime }$ : phantom; Case (1) $\mu_{\text{a}}$ : phantom, $\mu_{\text{s}}^{\prime }$ : real blood; Case (2) $\mu_{\text{a}}$ : real blood, $\mu_{\text{s}}^{\prime }$ : phantom; Case (3) $\mu_{\text{a}}$ : real blood, $\mu_{\text{s}}^{\prime }$ : real blood; Case (4) $\mu_{\text{a}}$ : real blood, $\mu_{\text{s}}^{\prime }$ : $\mu_{\text{s, ZrO2}}^{\prime }\times \frac{\mu_{\text{s, ZrO2}}^{\prime }(520\;\mathrm{nm})}{\mu_{\text{s, realBlood}}^{\prime }(520\;\mathrm{nm})}$ . All optical properties are shown in Fig. S4. ΔE values (color differences) between case 0 and the respective cases directly above the vein: (1) 2.6, (2) 0.8, (3) 2.1, (4) 2.7.

The aim of this section is to investigate how deviations in the optical properties of the blood phantom influence the characteristic blue appearance of venous vessel in the phantom. This study was motivated by the observation, described in Sec. 3.2, that the use of pigmented silicone to mimic the optical properties of blood leads to an increased $\mu_{\text{s}}^{\prime }$ due to intrinsic pigment scattering, resulting in values that are significantly higher than those expected for real blood, see Fig. 3. Our simulations aimed to quantify the impact of these optical properties on both the perceived color and the color difference (Δ*E*) at the skin surface directly above the vein. For this purpose, we modeled a vein with a depth of 1 mm and a diameter of 2 mm, as this configuration is one example that reliably produces the classic bluish hue, as illustrated in Fig. 4. The surrounding tissue was assigned the optical properties of the phantom material to ensure consistency with experimental conditions, see Fig. 2. As base simulation for the vein we used the reduced scattering and absorption coefficient for 70 % oxygenated blood of the phantom as shown in Fig. 5(case 0). We considered four distinct cases each representing a different combination of absorption and scattering coefficients for blood. The underlying optical properties are shown in Fig. S4. In the first case, the blood retained the absorption coefficient of the 70 % oxygenated blood phantom but adopted the reduced scattering coefficient of real blood, as depicted in Fig. 3(a). The second case involved blood with the absorption coefficient of real blood but the scattering coefficient of the phantom. The third case used both absorption and scattering coefficients corresponding to real blood, while the fourth case maintained the absorption coefficient of the phantom blood and combined it with the scattering coefficient of ZrO_2_ particles, which matches real blood at 520 nm. This should replicate the case that the coloring pigments do not have intrinsic scattering and we adjust the scattering by ZrO_2_ particles. The results reveal that the exchange of absorption alone, as in case two, has the least influence on the perceived color, yielding a Δ*E* of only 0.8. This value is barely perceptible to the human eye. In contrast, when both optical properties are set to those of real blood (case three), the color difference increases to a Δ*E* of 2.1, which is more noticeable. The most pronounced effect is observed when the reduced scattering coefficient is altered, either to that of real blood or to that of ZrO_2_ particles (cases one and four), resulting in Δ*E* values of 2.6 and 2.7, respectively. These values indicate a perceptible but not excessive color difference. Visual inspection of the simulated color impressions, as depicted in Fig. 5, confirms that an decreased $\mu_{\text{s}}^{\prime }$ compared to the phantom mimicking blood enhances the typical blue effect above the vein. However, even at the highest Δ*E* values, the difference remains within a range that does not compromise the practical utility of the phantom for visualizing the blue appearance of veins.

### 3.5. Silicone phantoms with blood vessels

**Fig. 6.**
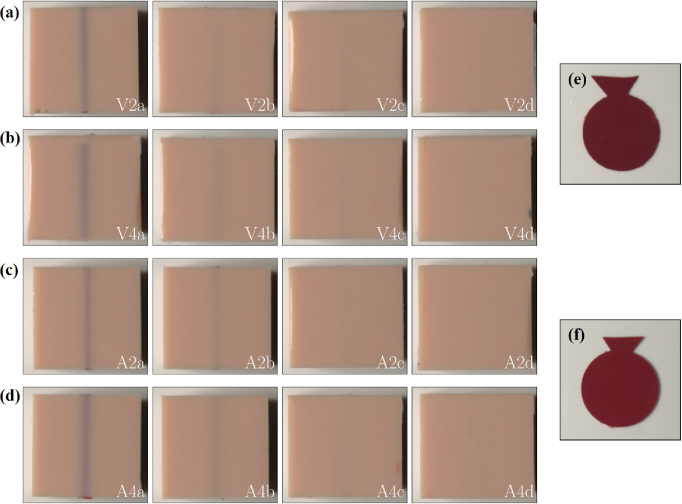
Photographs of all fabricated phantoms (a-d) with inclusion diameters of 2 and 4 mm at depths of 0.5, 1.0, 1.5 and 2.0 mm for both venous and arterial inclusions. The images were acquired using a standardized photo box and are labeled as follows: "V" for venous, "A" for arterial, "4" or "2" for the inclusion diameter, and "a", "b", "c" and "d" corresponding to depths of 0.5, 1.0, 1.5 and 2.0 mm, respectively. The cylindrical inclusion is placed in the center of the phantom. (e) Slab of silicone mimicking venous blood. (f) Slab of silicone mimicking arterial blood.

To experimentally validate the influence of vessel depth, diameter and oxygenation state, a series of tissue phantoms containing cylindrical inclusions was fabricated. These inclusions represented veins (70% oxygen saturation) and arteries (100% oxygen saturation). Venous inclusions were modeled with realistic geometries, while arterial inclusions — although less typical at the used depths — were included to systematically assess the effects of oxygenation and geometry. The cylindrical inclusions had diameters of 2 and 4 mm and were embedded at depths of 0.5, 1.0, 1.5 and 2.0 mm beneath the surface. These dimensions were chosen based on the typical diameters of visible veins [31,32]. The embedding depth was systematically varied within a reasonable range to ensure consistency and relevance [31,32]. An example of the mold used for fabrication is shown in Fig. 1. All phantoms were imaged using a standardized photo box setup, as detailed in Sec. 2.3, ensuring consistent and reproducibility experimental conditions. The optical properties of the phantoms correspond to those shown in Fig. 2 and Fig. 3. These produced phantoms are shown in Fig. 6(a-d). Figure 6(e) shows a slab of the pigmented silicone used for venous inclusions, while Fig. 6(f) shows a slab of the pigmented silicone used for arterial inclusions.

For both venous and arterial inclusions, vessels at depths of 0.5 and 1.0 mm were clearly visible. At larger depths (1.5 mm and 2.0 mm) the visibility decreases with depth. Notably, the color appearance of vessels located very close to the surface (0.5 mm) differ from those at 1.0 mm depth, reflecting the strong influence of vessel depth on the perceived color. At larger depths (1.5 mm and 2.0 mm), the characteristic blue coloration of the inclusions became nearly indistinguishable, irrespective of the oxygenation state. This trend was consistent across both inclusion diameters. However, larger vessels (4 mm in diameter) remained somewhat more discernible at a depth of 1.0 mm, particularly for venous inclusions. Close to the surface, differences in coloration between arterial and venous inclusions were observed. Venous inclusions appeared darker and more bluish, while arterial inclusions exhibited a more violet hue. These observations align with those reported by Kienle et al. [2]. At a depth of 1.0 mm, the characteristic bluish appearance of venous vessels was comparable to observations in real human tissue. Our results show that we were able to replicate the characteristic blue appearance of veins and demonstrate the visibility of arteries near the skin surface using our pigmented phantoms. However, venous and arterial vessels located deeper than 1.5 mm were difficult to detect with the naked eye. In real tissue, such vessels might still be visible due to the layered structure of skin and a less reduced scattering coefficient of the blood. A major difference between phantom and in vivo measurements lies in the surrounding medium outside the vessel. In the phantom experiments, a homogeneous medium is used modeling the optical properties of the skin. However, in vivo, subcutaneous fat is present at a depth of approximately 1 mm, where the penetration depth of light is larger due to lower scattering and absorption properties of subcutaneous fat compared to skin. As a result, deeper blood vessels can also be detected in vivo, even if the optical properties of the skin and blood were correctly modeled in the phantom. Consequently, even with accurate optical property matching, homogeneous phantoms may underestimate the depth and visibility of blood vessels compared to real, layered skin.

### 3.6. Comparison of simulated images and real photos

**Fig. 7.**
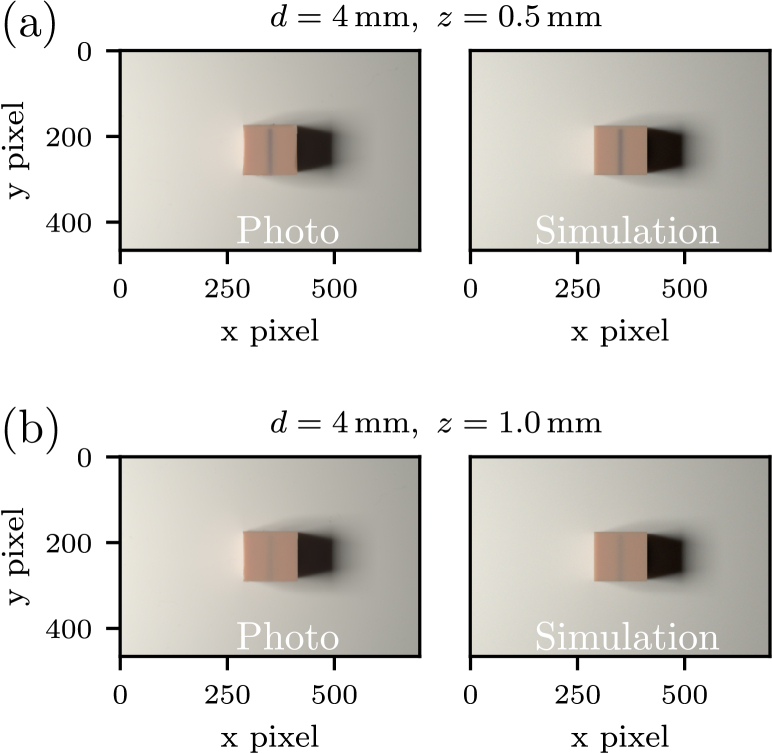
Comparison of experimental photographs (left) and corresponding simulation results (right) for vein phantoms with vessel depths of 0.5 and 1 mm and a diameter of 4 mm. The optical properties used in the simulation are based on the measured values of the phantoms (see Fig. 2 and Fig. 3). The color difference ΔE between simulation and real photo above the tissue is 1.2 for both cases. Above the vein, ΔE is 2.1 for case (a) and 2.2 for case (b).

To explore whether our phantoms are capable of replicating the results of the simulations, we performed simulations for venous vessel inclusions and compared them with corresponding photographs of physical phantoms. This approach allowed us to investigate the extent to which the predicted color phenomena and the measured optical properties align with the experimental observations. Each phantom contained a venous inclusion with a diameter of 4 mm, positioned at two distinct depths, 0.5 and 1.0 mm, beneath the surface, see Fig. 6(b). The optical properties for both tissue and blood were adopted from the phantom values presented in Fig. 2 and Fig. 3, respectively. These parameters were directly implemented in the simulation model to ensure consistency between the computational and experimental conditions. The physical phantoms were placed on a white silicone mat within the custom-designed light box, as described in Sec. 2.3. The simulation methodology and experimental protocol were analogue to Hevisov et al. [21]. The results of the experimental imaging are shown on the left and the corresponding simulation results are shown on the right of Fig. 7.

A direct comparison between the simulated and experimental images reveals a high degree of agreement in both the overall color and the appearance of the vein. For the phantom with the vein at *z* = 0.5 mm, Fig. 7(a), the observed color impression is more bluish. In contrast, the phantom with the vein at *z* = 1 mm exhibits the characteristic bluish appearance commonly associated with subcutaneous veins. For both veins depths, the tissue color in the simulated and experimental images is very similar, with a Δ*E* value of 1.2, indicating minimal deviations and consistent optical properties. When focusing specifically on the vein color, the visual appearance between simulation and experiment also exhibits a strong similarity. For a depth z of 0.5 mm, the Δ*E* value for the vein color is 2.1, while for *z* =  1.0 mm, the Δ*E* value is 2.2. On the one hand, this means that our phantoms closely match the specified parameters of optical size and geometry; on the other hand, it confirms that the visual effects of vein perception can be simulated using the light transport Monte Carlo method. These minor discrepancies between simulation and experiment may arise from several factors. Firstly, the optical properties of venous blood used in the simulation may not precisely correspond to those of the blood-mimicking material in the phantom. Specifically, between 400 and 600 nm, the values were obtained from measurements taken on very thin layers (60 µm), which could introduce slight inaccuracies. Second, the actual depth of the vein in the phantom may deviate slightly from the nominal values of 0.5 and 1 mm due to fabrication tolerances and the geometry of the phantoms themselves not being ideal. Additionally, geometric factors such as the alignment of the camera and the illumination source, as well as the precise positioning of the phantom within the light box, can influence the recorded color impression and contribute to residual differences between simulated and experimental results. Nevertheless, the overall close correspondence between the simulated and experimental images, especially regarding tissue color and the appearance of veins at both depths, underscores the validity our phantom approach.

### 3.7. Hyperspectral evaluation

**Fig. 8.**
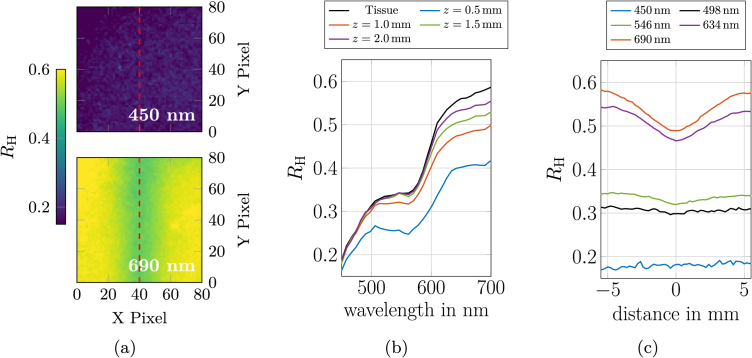
(a) Hyperspectral image of the tissue phantom with venous inclusion (*d* = 4 mm, *z* = 1 mm) at 450 and 690 nm. (b) Wavelength-resolved reflectance spectra measured directly above the vein for all depths (*z* = 0.5, 1, 1.5 and 2 mm), compared to the reflectance from surrounding tissue. (c) Cross-sectional reflectance profiles at selected wavelengths (450, 498, 546, 634 and 690 nm) for the phantom exhibiting a vein at *z* = 1 mm with *d* = 4 mm.

To quantify the influence of vessel depth on the reflected light over the vessel, hyperspectral imaging was performed using phantoms containing venous inclusions with a diameter of 4 mm at varying depths (*z* = 0.5, 1, 1.5 and 2 mm). These findings were used to explain the bluish appearance of veins. Corresponding photographs of the investigated phantoms are presented in Fig. 6(b). The hyperspectral setup, described in Sec. 2.5, was employed for all measurements. Each phantom was precisely positioned within the focal plane of the hyperspectral measurement system. The measured intensities of a phantom were referenced to a Spectralon white standard (Labsphere, USA) to calculate the reflectance *R*_H_. A pixel of a hyperspectral image has a size of 0.2x0.2 µm^2^. Figure 8(a) shows representative hyperspectral images at 450 and 690 nm of the phantom with a vein diameter of 4 mm and a depth of 1 mm. At 690 nm, the vein is clearly visible in the center of the image due to the change of *R*_H_ compared to the surrounding tissue, whereas at 450 nm, the vein is not discernible (red dashed line). This means that the reflected light at 450 nm did not interact with the vein. In Fig. 8(b), the wavelength-resolved reflectance acquired directly above the vein (red dashed line Fig. 8(a)) is shown for all investigated depths, in comparison to the reflectance, where no vein inclusion was present (X Pixel 80 ( Fig. 8(a)), which is 8 mm away from the vein). As expected, the data reveal a dependence of the measured reflectance on the vein depth. For the shallowest vein (*z* = 0.5 mm), the reflectance above the vein is markedly reduced across all measured wavelengths compared to the surrounding tissue. At a depth of 1 mm, the reflectance spectra above the vein closely match those of the tissue for wavelengths up to approximately 500 nm. Beyond this wavelength, a progressive decrease in reflectance is observed with increasing wavelength, reaching a maximum relative difference at 700 nm. This trend is further illustrated by the cross-sectional profiles shown in Fig. 8(c) for the phantom with vein depth at *z* = 1 mm. For larger vein depths (*z* = 1.5 and 2 mm), the reflectance spectra above the vein converge with those of the surrounding tissue for wavelengths up to 600 nm. Only between 600 and 700 nm, where tissue absorption is significantly lower, there is a systematic difference in the reflectance above the vein, as the light penetrates down to the vein and is partly absorbed by the vein. Figure 8(c) shows a cross-sectional analysis of the reflectance at selected wavelengths (450, 498, 546, 634 and 690 nm), providing spatially resolved profiles across the surface of the phantom with a venous cylindrical inclusion of 4 mm diameter at a depth of 1 mm. The largest relative changes in reflectance over the vein are observed in the red spectral region, while the blue region remains unaffected at this depth. This results are comparable to our in vivo measurements [2]. These findings indicate that, at a depth of 1 mm, blue light is predominantly scattered and absorbed by the superficial tissue layers, making it less sensitive to the presence of veins. In contrast, red light penetrates deeper into the tissue and is more strongly absorbed by the vein, resulting in relatively less reflected red light compared to the surrounding tissue. However, in absolute terms, more red light is reflected than blue light, compare Fig. 8(b,c). According to the Retinex theory, this difference in reflected light compared to the surrounding tissue leads to the perception of a bluish color, even though more red light is being reflected overall. Finally, at a depth of 0.5 mm, all wavelengths are reduced in comparison to the surrounding tissue, resulting in a slightly altered interpretation of the bluish color by the brain, which also can be seen in Fig. 6. This wavelength-dependent behavior aligns with our theoretical and experimental observations reported by Kienle et al. [2].

## 4. Discussion and conclusion

In this study, we conducted a systematic investigation into the visual appearance of blood vessels, analyzing how their depth, size and oxygenation state influence their visual perception. To achieve this, we combined experimental approaches with the support of light transport Monte Carlo simulations, ensuring a comprehensive and robust understanding of the observed phenomena.

Silicone phantoms with cylindrical inclusions, designed to simulate blood vessels, were developed to realistically mimic the optical properties $\mu_{\text{a}}$ and $\mu_{\text{s}}^{\prime }$ of skin, venous and arterial blood. The absorption and reduced scattering coefficient of the phantoms were adjusted using organic and inorganic pigments as well as ZrO_2_ particles. The silicone base was formulated to replicate the reflectance of the underarm region of Fitzpatrick skin type I. Vessel inclusions with varying depths and diameters were filled with pigmented silicone representing venous (70% oxygenation) and arterial (100% oxygenation) blood, approaching literature-based optical properties adjusted to 45% hematocrit.

Monte Carlo simulations were utilized to examine how deviations in the optical properties of tissue and blood vessel phantoms from literature values affect the perceived blue coloration of veins. The results indicate that such deviations in phantom optical properties do not substantially compromise the visual realism of the veins’ blue appearance. The resulting color differences (ΔE) remain within acceptable limits for visual assessment. To investigate the influence of vessel depth, diameter and oxygenation state, tissue phantoms with cylindrical inclusions representing veins and arteries were fabricated and analyzed. The inclusions varied in diameter (2 and 4 mm) and depth (0.5, 1.0, 1.5 and 2.0 mm). Photos were taken in a standardized photo box to ensure consistency. Results revealed that vessel visibility decreased with vessels depth. Vessels deeper than 1.5 mm were difficult to recognize. Close to the surface, veins appeared darker and more bluish, while arteries exhibited a violet hue. Larger vessels (4 mm) were more discernible than smaller vessels (2 mm).

Experimental validation was achieved by comparing real photographs of the silicone phantoms with simulated images. The agreement between real and simulated images supports the accuracy of the phantom design and the measured optical properties.

The origin of the bluish appearance of veins was investigated using hyperspectral imaging on a tissue phantoms containing a 4 mm diameter venous inclusion. The results revealed distinct reflectance behaviors above the vein, depending on the wavelength. At a vein depth of 1 mm blue light (450 nm) is predominantly scattered and absorbed by the superficial tissue layers, resulting in minimal interaction with the vein and actually no change in its reflectance above the vein. In contrast, red light (700 nm) penetrates deeper into the tissue and is strongly absorbed by blood in the vein. This absorption reduces the reflectance of red light above the vein significantly compared to the surrounding tissue. Importantly, above the vein, there is more red light reflected overall than blue light. However, in contrast to the surrounding tissue—where the same amount of blue light but a larger amount of red light is reflected—the vein appears bluish. This relative reduction in red reflectance above the vein, compared to its surroundings, creates the spectral contrast that leads to the bluish perception, as explained by Retinex theory [2]. At shallower depths (0.5 mm), reflectance above the vein across all wavelengths decreases, slightly altering the perceived bluish tone towards a violet tone. At depths of 1.5 and 2 mm the reflectance spectra above the vein converge with those of the surrounding tissue, making the vein increasingly less visible due to less contrast to the reflectance of the surrounding tissue. These findings align with theoretical evaluation and in vivo observations [2,6].

While the produced phantoms provide a valuable tool for visual evaluation, it is important to acknowledge some opportunities for refinement. For instance, the phantoms do not replicate the layered structure of skin or the wavelength-dependent scattering of real tissue. This results in altered light propagation and a deeper penetration depths, which in turn changes the visibility of vessels. It is recommended that future research also explore the role of melanin, given its significant effect on the absorption and scattering of visible light.

Phantoms are indispensable in biomedical optics, as they provide well-defined, reproducible optical properties for calibration, validation and inter-laboratory comparability of measurement systems [16,33]. While our phantoms were tailored for visual studies, advanced phantoms now enable quantitative benchmarking across modalities such as fluorescence, Raman and photoacoustic imaging [14,34–37]. Recent developments include multilayer and anatomically realistic constructs, as well as dynamic phantoms that simulate physiological processes like blood flow or metabolite diffusion [38–40]. These innovations allow for more comprehensive and application-specific device evaluation, supporting standardisation and regulatory acceptance. The development and use of such tunable and complex phantoms are essential for advancing biomedical optics and ensuring robust, equitable device performance.

In summary, the experimental investigation, supported by simulations, demonstrates that vessel depth, diameter and oxygenation are key factors influencing the visual appearance of blood vessels. The color and visibility of vessels are determined by the reflectance contrast with the surrounding tissue. The developed silicone phantoms provide an effective tool for studying vessel appearance, addressing the lack of systematic experimental investigations. Future work should investigate the role of melanin, refine phantom models for greater anatomical and optical accuracy and validate findings in vivo to enhance physiological relevance.

## Supplemental information

Supplement 1 SupplementalDocument https://doi.org/10.6084/m9.figshare.30620150

## Data Availability

Data underlying the results presented in this paper are not publicly available at this time but may be obtained from the authors upon reasonable request.

## Supplemental document

See Supplement 1 for supporting content.
