# Supplementary material for: Visual appearance of blood vessels: a phantom study
Source: Biomed Opt Express. 2026 Jan 29;17(2):1049–63. doi: 10.1364/BOE.579246 (PMC12904536; doi:10.1364/BOE.579246)
Supplement: Supplement 1 [file boe-17-2-1049-s001.pdf]

Supplemental Document

Biomedical Optics EXPRESS

Visual appearance of blood vessels: a phantom study: supplement

MARKUS WAGNER,[1,2,*] DAVID HEVISOV,[1,2] CORINNA KONRAD,[1,2] HANNES SCHMIDT,[1,2] FLORIAN FOSCHUM,[2] AND ALWIN KIENLE[1,2]

[1] *Faculty of Natural Sciences, Ulm University, D-89081 Ulm, Germany*
[2] *Institute for Laser Technologies in Medicine and Metrology at the University of Ulm, Helmholtzstr. 12, D-89081 Ulm, Germany*
[*] *markus.wagner@ilm-ulm.de*

This supplement published with Optica Publishing Group on 29 January 2026 by The Authors under the terms of the Creative Commons Attribution 4.0 License in the format provided by the authors and unedited. Further distribution of this work must maintain attribution to the author(s) and the published article's title, journal citation, and DOI.

Supplement DOI: https://doi.org/10.6084/m9.figshare.30620150

Parent Article DOI: https://doi.org/10.1364/BOE.579246

The visual appearance of blood vessels: a phantom study: supplemental document

1. OPTICAL PROPERTIES

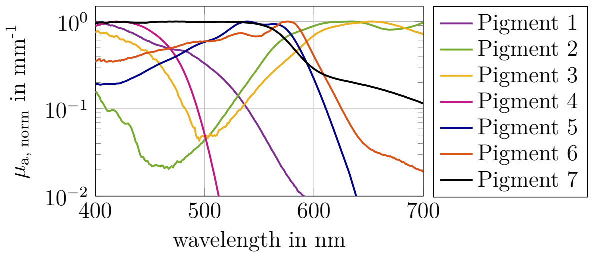

Fig. S1. The normalized absorption spectra of the seven used pigments for mimicking the tissue and blood absorption. The resulting names are shown in Tab. S1

Table S1. Pigments used in this study. Their corresponding absorption spectra are shown in Fig. S1.

Pigment	Name	Distributor
1	Eisenoxid Gelb-Orange	Kremer
2	Heliogen Blau Königsblau	Kremer
3	Heliogen Grün Dunkel	Kremer
4	Cromophtal® Yellow K 0990/K	BASF
5	Cinquasia® Pink K 4410	BASF
6	Cinquasia® Magenta K 4535/K	BASF
7	Eisen(III)-Oxid Pulver	Sigma-Aldrich

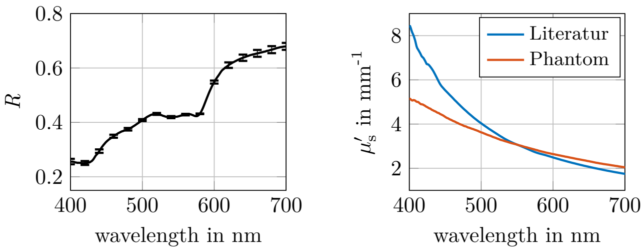

(a) (b) (c)

Fig. S2. (a) Measured reflectance spectrum of a human forearm, acquired using an integrating sphere setup. (b) The blue line shows a typical reduced scattering coefficient for skin reported by Bergmann et al. for porcine skin [1], while the orange line shows the reduced scattering coefficient of the zirconium dioxide particles used in the tissue-mimicking phantom. The ZrO_2 particle concentration was adjusted to match the skin value at 550 nm. (c) The theoretically calculated absorption coefficient was obtained by applying an inverse model to the integrating sphere reflectance data from (a) and the reduced scattering coefficients from (b). The blue line shows the absorption coefficient corresponding to the literature μ'_s (blue in (b)) and the orange line shows the theoretical μ_a derived using the μ'_s of the ZrO_2 particles (orange in (b)), which reproduces the reflectance of the human forearm (black in (a)).

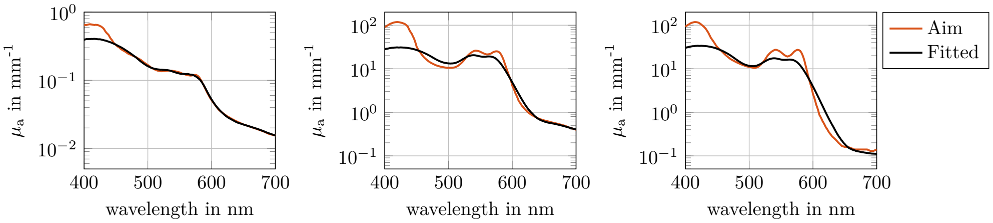

(a) (b) (c)

Fig. S3. The figure presents fitted absorption spectra (black lines) obtained by combining the pigments shown in Fig. S1, optimized to reproduce the target absorption spectrum (orange line). (a) Absorption spectrum of the tissue-mimicking base material. (b) Absorption spectrum corresponding to 70 % oxygenated blood. (c) Absorption spectrum corresponding to 100 % oxygenated blood.

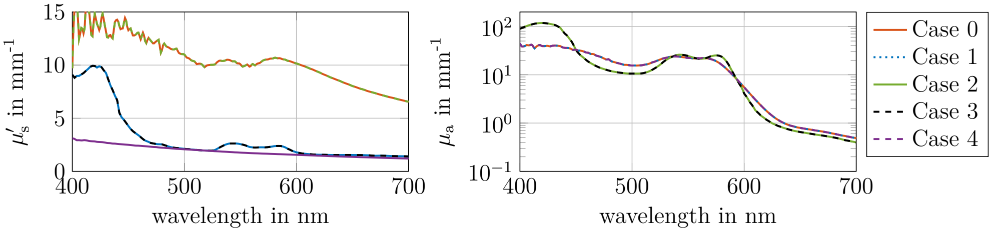

Fig. S4. Optical properties used for the simulated color impressions for different combinations of μ_a and μ'_s of blood: Case 0 (the real phantom): μ_a: phantom, μ'_s: phantom; Case (1) μ_a: phantom, μ'_s: real blood; Case (2) μ_a: real blood, μ'_s: phantom; Case (3) μ_a: real blood, μ'_s: real blood; Case (4) μ_a: real blood, μ'_s: $\mu'_{s,\,ZrO2} \times \frac{\mu'_{s,\,ZrO2}(520\,\text{nm})}{\mu'_{s,\,realBlood}(520\,\text{nm})}$.

REFERENCES

1. F. Bergmann, F. Foschum, L. Marzel, and A. Kienle, "Ex vivo determination of broadband absorption and effective scattering coefficients of porcine tissue," in *Photonics,* vol. 8 (MDPI, 2021), p. 365.
